# Expression of Lactate Dehydrogenase in *Aspergillus niger* for L-Lactic Acid Production

**DOI:** 10.1371/journal.pone.0145459

**Published:** 2015-12-18

**Authors:** Khyati K. Dave, Narayan S. Punekar

**Affiliations:** Department of Biosciences and Bioengineering, Indian Institute of Technology Bombay, Mumbai, Maharashtra, India; USDA Forest Service, UNITED STATES

## Abstract

Different engineered organisms have been used to produce L-lactate. Poor yields of lactate at low pH and expensive downstream processing remain as bottlenecks. *Aspergillus niger* is a prolific citrate producer and a remarkably acid tolerant fungus. Neither a functional lactate dehydrogenase (LDH) from nor lactate production by *A*. *niger* is reported. Its genome was also investigated for the presence of a functional *ldh*. The endogenous *A*. *niger* citrate synthase promoter relevant to *A*. *niger* acidogenic metabolism was employed to drive constitutive expression of mouse lactate dehydrogenase (*mldhA*). An appraisal of different branches of the *A*. *niger* pyruvate node guided the choice of *mldhA* for heterologous expression. A high copy number transformant C12 strain, displaying highest LDH specific activity, was analyzed under different growth conditions. The C12 strain produced 7.7 g/l of extracellular L-lactate from 60 g/l of glucose, in non-neutralizing minimal media. Significantly, lactate and citrate accumulated under two different growth conditions. Already an established acidogenic platform, *A*. *niger* now promises to be a valuable host for lactate production.

## Introduction

Lactic acid is a versatile organic acid used in various industrial applications. It is a commonly used acidulant and preservative in food, leather, textile industries and helps in controlled drug delivery. Demand for lactic acid has increased in recent years due to two emerging products—polylactide (a biodegradable plastic) and ethyl lactate (an environment friendly solvent). The L(+) form of lactate is used in food and pharma industries as humans can assimilate this isomer [1]. Industrial scale biosynthesis of L-lactic acid is achieved through fermentation of microorganisms belonging to *Lactobacillus*, *Bacillus* and *Rhizopus* genera. *Lactobacillus* species were the first promising candidate for lactic acid production. They are fastidious with respect to growth requirements and cannot utilize starchy raw materials. Hence the pretreatment of raw materials raises the cost of production. Their growth is inhibited at low pH during lactate fermentation and the addition of neutralizing agents makes downstream processing difficult [1]. Amongst filamentous fungi *Rhizopus oryzae* is known to naturally produce lactate. But lactate production using this fungus requires near neutral pH conditions and yields are compromised due to formation of ethanol and fumarate as by-products [2,3]. Different organisms have been engineered to improve process parameters and increase lactate yields. Among fungi, *Saccharomyces cerevisiae* [4–6], *Kluyveromyces lactis* [7,8], *R*. *oryzae* [9], *Pichia stipites* [10], *Candida utilis* [11] and *Candida sonorensis* [12] were genetically modified to produce L-lactate. Lactic acid production by most engineered hosts is hampered by the formation of high levels of by-products like ethanol (in yeast) and fumarate (in *Rhizopus*), compromised growth due to gene deletions, low pH tolerance, long fermentation time and inability to utilize different raw materials [1,13]. Thus, development of other production platforms to overcome some of these limitations continues to attract attention.

*Aspergillus niger* produces high levels of citrate and its acidogenic fermentation parameters are well established. Its saprophytic mode of nutrition allows for utilization of various raw materials and grows well at pH 3.0 or lower. These advantages along with GRAS (Generally Regarded as Safe) status make this organism an industrial favorite to produce citrate and gluconate [14]. With the advent of different genetic tools, *A*. *niger* has also been manipulated to produce oxalate [15], succinate [16] and itaconate [17–20]. *A*. *niger* is not a natural L-lactate producer and a functional lactate dehydrogenase (LDH) has not been reported in this fungus. Recently, two different *Aspergillus* species, albeit with indistinct acidogenic capacities, were engineered to produce lactate [21,22]. However, *A*. *niger* is a well known acid producer with better understood metabolic profile and a strong glycolytic flux [23]. The pyruvate so formed could be diverted to form L-lactate by expressing a suitable *ldh* gene. We chose and expressed the mouse LDH using a homologous strong constitutive *A*. *niger* citrate synthase promoter [24]. Such a transformant was capable of producing L-lactate aerobically on minimal media (Indian Patent Application No. 2077/MUM/2014). The advantages of *A*. *niger* as a lactate production platform are highlighted in this report.

## Material and Methods

### Strains and media

*A*. *niger* NCIM 565 (National collection of Industrial Microorganisms, National Chemical Laboratory-Pune, India) was the parent strain for this study. Different *A*. *niger* strains were maintained on potato dextrose (PDA) agar or yeast nitrogen base (without amino acids and ammonium sulfate), dextrose and ammonium nitrate (YDA) agar supplemented with DL- phosphinothricin (PPT) (YDA+PPT agar for *bar* transformants). The PPT (technical grade sample from Bayer Crop-Science; supplied as 45–50% aqueous solution, w/v) at 1.0 mg/ml of final concentration was used for selection of transformants. The *bar* marker from plasmid pCB1265 was employed to select *A*. *niger* transformants. *A*. *niger* mycelia were harvested from shake flask cultures grown in minimal medium (MM) [25] for protein extraction and lactate fermentation. The MM contained 20.0 g/l glucose, 3.0 g/l KH_2_PO_4_, 6.0 g/l Na_2_HPO_4_, 0.5 g/l MgSO_4_.7H_2_O, 2.25 g/l NH_4_NO_3_, 10 mg/l ZnSO_4_.7H_2_O, 3.0 mg/l MnSO_4_.7H_2_O, 1.5 mg/l Na_2_MoO_4_.H_2_O, 20.0 mg/l FeCl_3_.6H_2_O and 1.0 mg/l CuSO_4_.H_2_O. The medium pH was adjusted with 0.1 N HCl to 5.5–6.0. For citrate production, *A*. *niger* growth was carried out in acidogenic medium (AM) containing 140.0 g/l sucrose, 1.0 g/l KH_2_PO_4_, 0.1 g/l Fe(NH_4_)_2_(SO_4_)_2_, 2.25 g/l NH_4_NO_3_ and 0.25 g/l MgSO_4_.7H_2_O for eight days. *A*. *niger* strains were also grown in MM under conditions of hypoxia according to Terabayashi et al. [26].

### Construction of heterologous and homologous *ldh* expression vectors

Mouse *ldh*A cDNA (NCBI accession number BC094019) was PCR amplified from a cDNA clone (pCMV from OriGene, USA), using the primers ldhNcF1 and ldhXbR1 (Table 1). The amplicon (999 bp DNA encoding a 332 residue protein) was cloned between *Nco*I and *Xba*I sites in pCBΔXCE [24] by replacing the EGFP CDS. The resultant plasmid pCBΔXCmldh consisted of *PcitA*-*mldhA* expression cassette along with the *bar* selection marker. The putative *A*. *niger ldh* sequence (corresponding to An04g08220 of *A*. *niger* CBS strain) was PCR amplified (primers AnldhNcF1 and AnldhXR1; Table 1) from *A*. *niger* NCIM 565 genomic DNA. This amplicon was cloned between *Nco*I and *Xba*I sites of pCBΔXCmldh thereby replacing the mouse *ldh*A cDNA to obtain pCBΔXCAnldh.

**Table 1:** List of primers used in this study.

Primer	Sequence (5'-3')^a^	Description
ldhNcF1	ccca**ccatgg**caaccctcaaggacc	Mouse *ldhA* specific forward primer (*Nco*I site)
ldhXbR1	gg**tctaga**ttagaactgcagctccttctgg	Mouse *ldhA* specific reverse primer (*Xba*I site)
AnldhNcF1	gaga**ccatgg**caacaatcgccttaatcg	*A*. *niger ldhA* specific forward primer (*Nco*I site)
AnldhXR1	gc**tctaga**ctacgaccctccagcatca	*A*. *niger ldhA* specific reverse primer (*Xba*I site)
CitF4	ctaccgtgtcctgatataccc	*A*. *niger PcitA* specific forward primer
mldhR4	caactgtaatcttgttctggg	Mouse *ldhA* reverse primer
actAF	ttgcggtacagcctccattg	*A*. *niger actA* forward primer
actAR	cgcttggactgtgcctcatc	*A*. *niger actA* reverse primer

^a^Restriction enzyme recognition sites are in bold.

PCR reactions in 100 μl contained—3.5mM MgCl_2_, 250 μM dNTP’s, 0.5 μM primers and 5 units of *Pfu* polymerase (MBI Fermentas, St. Leon-Rot, Germany). Cloning and propagation of plasmids was done in *Escherichia coli* XL1-Blue (Stratagene, CA, USA), according to standard procedures [27].

### *A*. *niger* transformation and characterization of transformants

*A*. *niger* was transformed and the *A*. *niger* transformants were selected on YDA+PPT agar [24]. Genomic DNA was isolated from mycelia using QIAGEN DNeasy Plant Mini kit. *PcitA-mldhA* copy number in the genome of *A*. *niger mldh* transformants was determined by qPCR (Stratagene Mx3000P; Agilent Technologies). The *PcitA-mldhA* fragment was amplified with CitF4 and mldhR4 primer pair (Table 1). The single copy *A*. *niger* actin gene served as a reference and was amplified with actAF and actAR primers (Table 1). Amplification efficiencies were determined using serial dilutions (0.1–10 ng/μl) of genomic DNA. The qPCR reactions (in 20 μl) were performed in triplicates and each reaction mixture contained 10 μl 2× Brilliant III Ultra fastSYBR Green master mix (Agilent Technologies), 3 μl suitably diluted genomic DNA and 50 nM each of forward and reverse primers. The qPCR conditions were as follows: initial denaturation at 95°C for 3 min followed by 40 cycles of 95°C for 20 s, 61°C for 30 s. Melt curve analysis was performed to check for the presence of primer dimers and/or nonspecific products. The copy number was calculated according to Skulj et al. [28].

### cDNA preparation

Total RNA from *A*. *niger* was prepared using QIAGEN RNeasy Plant Mini kit. *A*. *niger ldhA* cDNA was prepared from total RNA using Transcription first strand cDNA synthesis kit (Roche Diagnostics India Pvt. Ltd.). Oligo(dT) primers were used to generate cDNA which was subsequently purified using cDNA purification column (Invitrogen). The amplification of *A*. *niger ldhA* cDNA was done with gene specific primers AnldhNcF1 and AnldhXR1.

### Preparation of cell-free extract and lactate dehydrogenase assay

*A*. *niger* mycelial proteins were extracted with 2 volumes (cell wet weight:volume) of ice-cold extraction buffer (200 mM Tris-HCl pH 8.0, 1 mM 2-mercaptoethanol and 1 mM phenylmethanesulfonylfluoride). The cell-free extract was clarified at 12400×g for 20 min in a refrigerated centrifuge. For LDH assays, the final supernatant was desalted (on Sephadex G-25 column) before use.

The LDH activity was determined in the direction of lactate oxidation, by measuring the NADH formed at 340 nm. The assay mixture composed of 100 mM Tris-HCl buffer (pH 8.0), 200 mM sodium L-lactate, 2.5 mM NAD^+^ and suitably diluted *A*. *niger* cell-free extract. One unit of enzyme activity corresponds to the amount of enzyme that generates 1.0 nmole of NADH per min. The LDH activity was also monitored in the direction of pyruvate reduction (1.0 mM sodium pyruvate, 0.1 mM NADH in the same buffer as mentioned above, but at pH 7.4), when required. The LDH activity staining protocol was essentially as that for glutamate dehydrogenase [25], but using lactate and NAD^+^ as substrates.

### Analytical methods

L-Lactate, citrate and glucose were estimated enzymatically. The assay mixture for lactate estimation consisted of 1.0 M glycine buffer (pH 9.5), 0.4 M hydrazine, 2.5 mM NAD^+^, 10 units of rabbit muscle L-LDH (Sigma Aldrich) and a suitable aliquot of the lactate sample. The mixture was incubated at 37°C for 60 min and absorbance at 340 nm was recorded. Appropriate enzyme blanks were included and L-lactate concentration was calculated using a standard curve. The lactate concentration was determined in millimolar (mM) units and 1 mM corresponds to 0.09 g/l. Citrate was estimated using citrate lyase (from *Aerobacter aerogenes*; Roche Diagnostics India Pvt. Ltd.) based on established procedure [29]. After an initial 15 min incubation with phenylhydrazine, the citrate sample was incubated for further 15 min with citrate lyase assay buffer. The absorbance of oxaloacetate phenylhydrazone was measured at 330 nm. Appropriate enzyme blanks were used to account for endogenous oxaloacetate contribution. Glucose was estimated using glucose oxidase-peroxidase reagent (Biolab Diagnostics Pvt. Ltd., India).

For intracellular lactate and citrate estimations, the *A*. *niger* mycelia were harvested by filtering through muslin cloth, washed with 3–5 volumes of distilled water, blotted dry and frozen immediately in liquid nitrogen. The whole operation was concluded within couple of minutes after harvest. The mycelia were crushed in liquid nitrogen and suitable buffer to obtain cell-free extracts. The cell-free extracts were treated with perchloric acid: methanol: water (8:40:52) mixture (10% of sample volume) to precipitate proteins and the precipitate cleared by centrifugation (10000×g for 5 min). Clear supernatants were neutralized with 1.0 M KOH before estimating the lactate/citrate present. The protocol employed here takes a few minutes and may not rapidly quench the mycelial metabolism [30]; to this extent, intracellular lactate and citrate measurements may be biased. However, their extracellular levels were better assessed as the samples were quickly filtered to remove mycelia and frozen. Intracellular concentration of lactate/citrate was calculated assuming an intracellular volume of 0.233 ml/g wet weight of *A*. *niger* mycelia [31].

Unless otherwise mentioned, all enzyme activity data and the metabolite measurements are representative of at least three separate experiments (performed in duplicates). Error bars represent the standard deviation.

### Shake flask culture for lactate fermentation

The *A*. *niger* NCIM 565 and *ldhA* transformants were grown in MM supplemented with different glucose concentrations (1–10 percent). CaCO_3_ (30 g/l) was added to MM for growth under neutralizing conditions. Different *A*. *niger* strains were inoculated (10^8^ spores/100 ml) in 200 ml medium (in 1000 ml Erlenmeyer flasks) and the lactate levels were monitored for 7 days.

## Results and Discussion

### Search for a functional *ldh* locus in *A*. *niger*

Use of *A*. *niger* as a host to produce lactate requires that an endogenous or heterologous *ldh* gene be expressed. Presence of a functional LDH is not reported in *A*. *niger*. Marginal but reproducible lactate oxidation activity was observed in the parent strain suggesting that an endogenous LDH-like enzyme may be present in *A*. *niger*. Lactate (in μM range) was also detected in the culture media under hypoxic growth of a few Aspergilli [26]. Putative *ldh* loci were identified from *in silico* analysis of published genome sequences of Aspergilli, including *A*. *niger* [32,33]. In this context, functional annotation of *A*. *niger ldh* and exploring its role in L-lactate formation is important. Five out of 272 hits in *A*. *niger* ATCC 1015 JGI genome database (v4.0) are annotated as putative NAD-dependent lactate/malate dehydrogenase (Table 2); others are either putative cytochrome-dependent dehydrogenases or hypothetical proteins [34]. A blastp analysis, with three different *ldhA* sequences (*R*. *oryzae*, AAF74436.1; *A*. *nidulans*, AN5842; mouse, BC094019) as queries, also retrieved one of these five putative *ldh* from JGI genome database (JGI protein id: 1094595, Table 2). Further, this sequence corresponds to An04g08220 locus (putative *ldhA*) in *A*. *niger* CBS genome [32,33]. This putative *ldhA* sequence contains one intron and shows around 30% amino acid sequence similarity with *R*. *oryzae* LDH (isoform A). The NADH binding sites are conserved in this putative *ldh* from *A*. *niger*; but the LDH signature sequence at the active site (VGV**R**DSES) is not well conserved. The catalytically important H residue is replaced by an R residue. This active site His (H193 of mouse LDH) is conserved in all functional LDHs [35]. This residue is also conserved in putative LDH from other Aspergilli except for *A*. *niger* and *A*. *fumigatus* (S1 Fig). The other putative NAD-dependent lactate/malate dehydrogenases (Table 2) do contain this H residue. However, a comparison of their full length protein sequences suggests they may be malate dehydrogenases and hence they were not considered.

**Table 2:** Putative *ldh* sequences found in *A*. *niger* ATCC 1015 JGI genome database.^a^

JGI protein id	Location	Genbank (CBS strain)	Putative role	Similarity	
				*R*. *oryzae* LDH	Mouse LDH
1143375	chr_304:32072–33915 (-)	CAK42176.1	NAD/NADP lactate/malate dehydrogenase	Score: 50.1 Identity: 24% Coverage: 93%	Score: 66.2 Identity: 27%Coverage: 96%
48047	chr_701:1791847–1793109 (-)	CAK40769.1	NAD/NADP lactate/malate dehydrogenase	Score: 43.9 Identity: 24% Coverage: 68%	Score: 43.5 Identity: 24% Coverage: 87%
1094595	chr_603:61034–62047 (-)	CAK44788.1	NAD/NADP lactate/malate dehydrogenase	Score: 165 Identity: 36% Coverage: 91%	Score: 105 Identity: 27% Coverage: 90%
1143842	chr_401:461235–462893 (-)	CAK39307.1	NAD/NADP lactate/malate dehydrogenase	Score: 61.6 Identity: 24% Coverage: 96%	Score: 55.5 Identity: 25% Coverage: 91%
1156951	chr_301:246807–247963 (+)	CAK39136.1	NAD/NADP malate/lactate dehydrogenase	Score: 15.4 Identity: 31% Coverage: 19%	Score: 16.2 Identity: 57% Coverage: 1%

^a^ JGI protein models were identified by searching the *A*. *niger* ATCC 1015 JGI genome database (v4.0) with the keyword ‘L-lactate dehydrogenase’ and the putative NAD-dependent lactate/malate dehydrogenase were identified based on NAD/NADP binding domains.

A functional annotation of the putative *ldhA* ORF (JGI protein id: 1094595) was attempted. The genomic clone, containing a predicted 90 nt intron and encoding a 308 residue protein, was fused to the *A*. *niger* citrate synthase promoter (*PcitA*) (pCBΔXCAnldh) for constitutive expression in *A*. *niger*. Around twenty transformants (through *bar* selection) were obtained and the integration of *PcitA-Anldh* cassette was confirmed by genomic PCR. None of these transformants displayed significant increase in their LDH activity (activity was comparable to that of the parent strain). The functionality of this ORF was also tested by expressing the corresponding cDNA in *E*. *coli*. For this, cDNA was prepared from one of the *A*. *niger* transformants (the intron was spliced as expected), cloned in pET-28a and expressed in *E*. *coli* BL21 (DE3). However, the expressed protein was not active on different substrate combinations like L-lactate, D/L-lactate, ethanol, NAD^+^ and NADP^+^ (data not shown). An R174H site directed mutant was constructed to see if this made the putative *A*. *niger* LDH functional. This mutant protein also did not show any LDH activity. The putative *ldhA* in *A*. *niger* (JGI protein id: 1094595) therefore does not code for a functional LDH in *E*. *coli*.

### Expression of heterologous mouse *ldhA (mldhA)* in *A*. *niger*

**Fig 1:**
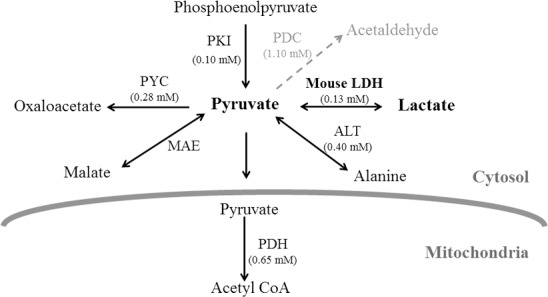
Metabolic fates of pyruvate in *A*. *niger*. All enzymes catalyzing the reactions with pyruvate (as substrate or product) are depicted and the pathway shown with dotted arrow is from *A*. *nidulans*. ALT- alanine transaminase, LDH- lactate dehydrogenase [40], MAE- malic enzyme [41], PDC- pyruvate decarboxylase, PDH- pyruvate dehydrogenase complex, PKI- pyruvate kinase [42] and PYC- pyruvate carboxylase [43]. Respective *K*_*m*_ values for the enzymes are given in brackets. These are either taken from *Aspergillus* literature or for the corresponding yeast enzymes from BRENDA (www.brenda-enzymes.org).

Since attempts to identify functional *ldh* gene(s) from *A*. *niger* genome were unsuccessful, a heterologous *ldh* sequence was expressed to divert the carbon flux from pyruvate towards lactate in *A*. *niger*. A range of *ldh* sources have been evaluated for fungal lactate production since the optimal carbon distribution at the pyruvate node is dependent upon the properties of LDH employed [12,36,37]. In the earlier two reports with Aspergilli, bovine and *R*. *oryzae ldhA* genes were used to achieve LDH expression [21,22]. However, the *A*. *oryzae* strains constructed to express *R*. *oryzae ldhA* genes did not accumulate detectable amounts of lactate [21]. Expressing LDH with appropriate kinetic features is desirable for better lactate yields. Different branches of the *A*. *niger* pyruvate node were scrutinized and the kinetic and regulatory properties of different enzymes at this node were analyzed. With micromolar intracellular pyruvate levels (40 μM, [30]) and correspondingly low Km values for different enzymes (Fig 1), choice of an LDH with low Km for pyruvate and high K_i_ for lactate was obvious. Accordingly, mouse *ldhA* (*mldhA*) having low *K*_*m*_ for pyruvate (0.13 mM) was chosen for expression. Promoters of glycolytic pathway enzymes namely, *Ppki* and *PgpdA* were employed earlier to manipulate oxalate and itaconate production in *A*. *niger*, respectively [15,20]. However, *ldh* expression for lactate production in fungi was earlier achieved through various promoters like yeast *PDC1* [11,36], *A*. *oryzae sodM* [21] and *A*. *nidulans gpdA* [22] genes. The fact that *A*. *niger* is a major citrate producer with a high glycolytic flux makes its own citrate synthase promoter (*PcitA*) an ideal pick. The strong constitutive *A*. *niger PcitA* was also selected for as it was active throughout growth on MM and AM [24]. The compact *PcitA* (0.5 kb) was used successfully to express both homologous and heterologous CDSs in *A*. *niger* [24,38]. Further, it is comparable in strength to the well established promoters like *PglaA* and *Pgpd* [39]. Not surprisingly, the choice of *mldhA* in combination with *PcitA* for LDH expression resulted in good titers of lactic acid (see below).

The plasmid pCBΔXCmldh (bearing *PcitA*-*mldhA* gene construct, see Material and methods section) was linearized and used to transform *A*. *niger* NCIM 565. Most of the *bar* ^+^ transformants displayed significantly higher LDH specific activity (in cell-free extracts) than the parent strain. No extracellular (spent medium) LDH activity was found in any of these transformants. The pyruvate reduction rates were always higher than the corresponding lactate oxidation rates; lactate oxidation is a better measure of specific LDH activity. Negligible yet consistent lactate oxidation activity was observed in the parent strain; that the rudimentary activity may be due to endogenous LDH cannot be excluded at this point. That *A*. *niger* may contain an endogenous LDH (or LDH-like) activity is discussed below.

**Fig 2:**
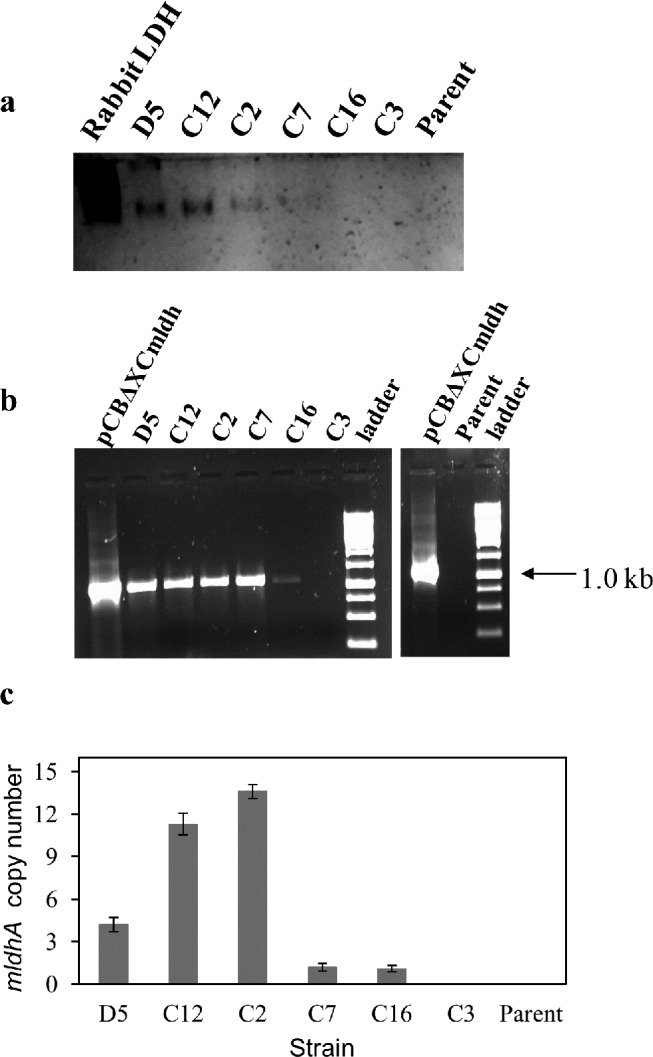
Analysis of *mldhA* transformants by activity staining, genomic PCR and qPCR: a—Activity staining for LDH from *A*. *niger* and six of its transformants. Desalted cell-free extract (5 μg protein each) was loaded on polyacrylamide gels. Purified rabbit skeletal muscle LDH served as positive control. b—PCR amplification of integrated *PcitA- mldhA* DNA from the transformants. Parent *A*. *niger* genomic DNA and pCBXCmldh served as negative and positive controls, respectively.c—*PcitA*-*mldhA* copy number determination in transformants by qPCR. The *mldhA* copy number was determined using the single copy *A*. *niger* actin gene as reference. The data are plotted as mean values obtained from different concentrations of genomic DNA. Error bars represent the standard deviation.

Six *bar* ^+^ transformants displaying a range of LDH specific activities (two highest- D5, C12; two intermediate- C2, C7; and two basal- C16, C3) were selected for further analysis (Table 3). The enzyme activity staining results were consistent with the corresponding LDH specific activities measured for these six transformants (Fig 2A). The integration of *PcitA*-*mldhA* DNA into the genome of the transformants was confirmed by genomic PCR. A 1.0 kb amplicon (with primers citF4 and ldhXR1) was obtained for all the transformants except C3 (Fig 2B); this is consistent with the corresponding LDH activity data (Table 3). Integrated DNA could also be picked up in Southern blots (not shown). Since the DNA integration events are random, the observed differences in LDH specific activity of the transformants may reflect either copy number effects or locus specific transcriptional effects. Therefore, *PcitA-mldhA* copy number in the genome of these *A*. *niger* transformants was determined by qPCR (Fig 2C). All the transformants (except C3 strain, which was however *bar* ^+^) had at least one copy of *PcitA*-*mldhA* cassette integrated in their genome (Table 3). There was no linear correlation between the copy number integrated and LDH specific activity. Transformants with only one copy of *PcitA*-*mldhA* integrated (C7 and C16 strains) had different LDH specific activities. The D5 transformant with 4 copies of *PcitA-mdlhA* showed higher LDH specific activity than C12 and C2 transformants having 11 and 13 copies of *PcitA-mdlhA*, respectively. This could possibly be the effect of integration context on expression levels. While marginal LDH (lactate oxidation) activity was observed in the parent strain, as expected, it was devoid of the *PcitA*-*mldhA* cassette. Similar analysis in the case of *A*. *oryzae ldh* transformants is not available [21]. The LDH activity in *A*. *brasiliensis ldh* transformants increased with *ldhA* gene copy number up to 6 copies. But LDH activity gradually decreased as the gene copy increased above 6 [22].

**Table 3:** LDH specific activity of the *A*. *niger mldhA* transformants.

Strain	Activity (U)^a^	Specific activity (U/mg protein)	*mldhA* copy number
D5	360.5 ± 35.7	171.7 ± 15.0	4
C12	302.7 ± 14.4	137.0 ± 14.0	11
C2	180.2 ± 31.2	81.3 ± 6.5	13
C7	86.7 ± 16.8	40.0 ± 2.0	1
C16	18.8 ± 6.9	8.3 ± 3.1	1
C3	2.0 ± 1.1	0.9 ± 0.7	0
Parent	4.8 ± 4.6	2.3 ± 1.5	0

^a^ LDH was assayed in the direction of lactate oxidation.

### Lactate formation in *mldhA* transformants

**Fig 3:**
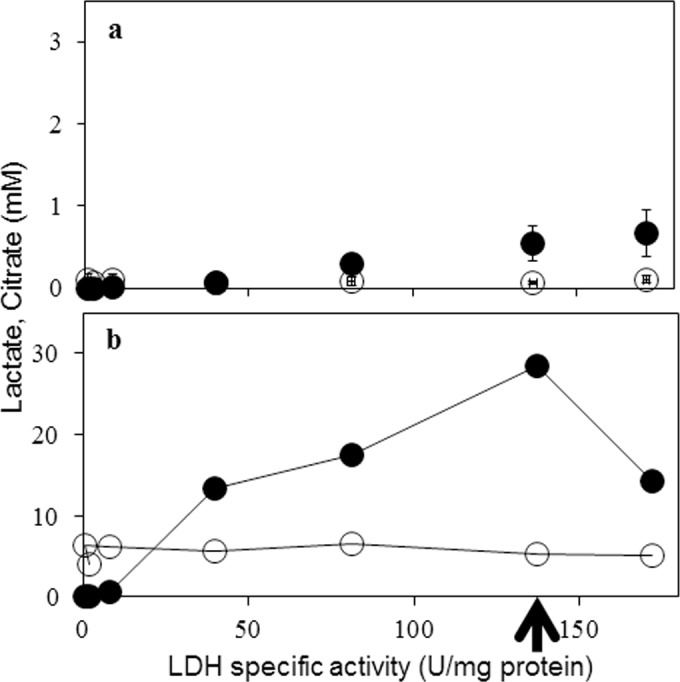
LDH specific activity and lactate and citrate levels in *mldhA* transformants: Extracellular (Panel a) and intracellular (Panel b) concentrations of L-lactate (filled circles) and citrate (open circles) are shown. Data in panel b is an average of two measurements. Each LDH specific activity data point represents one individual transformant (see Table 3). Arrow indicates the data for C12 strain.

The parent *A*. *niger* NCIM 565 strain is incapable of making L-lactic acid and if any, sub-micromolar levels of lactate are detected in its cell-free extracts. With the exception of C3 strain, L-lactate was detected in the spent medium (extracellular) of all the transformants grown in MM (without pH regulation) for 24 h (Fig 3). Intracellular lactate (in the cell-free extracts) was also detected in these transformants and the levels were invariably higher than those found in the spent medium. The levels of citrate in all the transformants however remain unchanged. In general, lactate levels were positively correlated with the LDH specific activity (Fig 3), and not the *mldhA* copy number (see Table 3), of the transformants. The transformant D5 is an exception in that, despite the highest LDH specific activity (albeit with only 4 gene copies integrated), it had lower lactate levels when compared with C12 strain. Strain D5 also conidiated poorly on PDA plates. Increasing LDH specific activity in transgenic *S*. *cerevisiae* [37], *C*. *sonorensis* [12] and *A*. *brasiliensis* [22] had a positive impact on lactate yield. The C12 transformant strain of *A*. *niger* showed maximal extracellular lactate levels on MM containing 1.0 percent glucose (Fig 3). This transformant is deposited in the Microbial Culture Collection at NCCS, Pune, India (Accession number MCC0019). The C12 strain was further tested under different growth conditions for lactate production.

### Lactate formation by the transgenic *A*. *niger* C12 strain

#### Growth on different glucose concentrations

**Fig 4:**
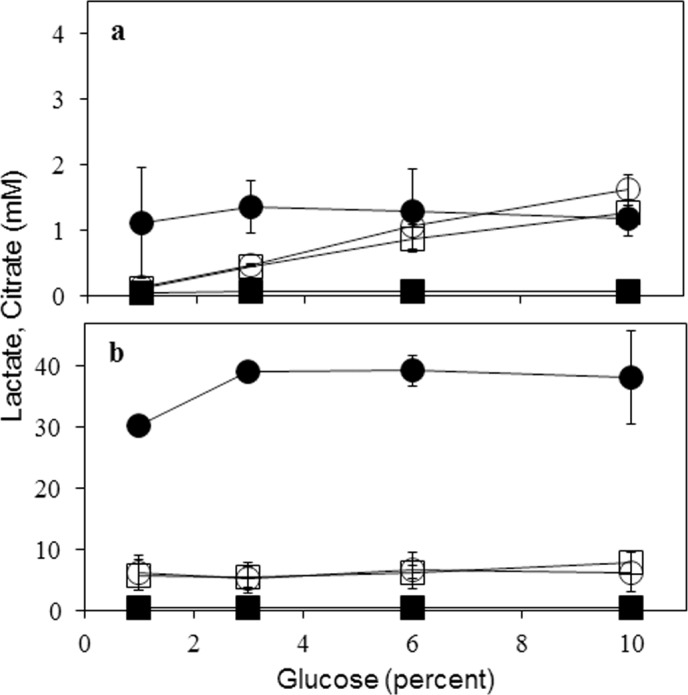
Effect of increasing medium glucose on the formation of lactate and citrate by *A*. *niger* C12 strain: Extracellular (Panel a) and intracellular (Panel b) concentrations of the two acids from C12 strain (circles) and the parent (squares) are shown. Filled symbols represent L-lactate and open symbols represent citrate. Data are for 24 h growth.

The effect of increasing initial glucose concentrations on lactate production by the C12 strain was studied. The LDH specific activity in this strain was not significantly different (range 130–180 U/mg protein) when grown for 24 h on MM with different glucose amounts (1 to 10 percent); whereas the corresponding LDH specific activity of the parent strain ranged between 8–17 U/mg of protein. Lactate was detected both in the spent medium (extracellular) and in the cell-free extracts (intracellular) of C12 strain (Fig 4). The lactate levels were unaffected by increased glucose in the growth medium. Intracellular lactate levels after 24 h growth were invariably higher (between 30–40 mM) than those found in the spent medium (around 1.5 mM). It was also instructive to measure citrate levels as *A*. *niger* is a recognized citrate producer. While the intracellular citrate remained steady (between 7–9 mM), its levels increased in the spent medium with increasing glucose; similar results for citrate were obtained with the parent *A*. *niger* strain (Fig 4). Detection of TCA cycle intermediates (malate and succinate) were reported with transgenic *A*. *oryzae* and it was suggested that excess glucose may induce pyruvate run-off into the TCA cycle [21].

#### Lactate fermentation on MM

**Fig 5:**
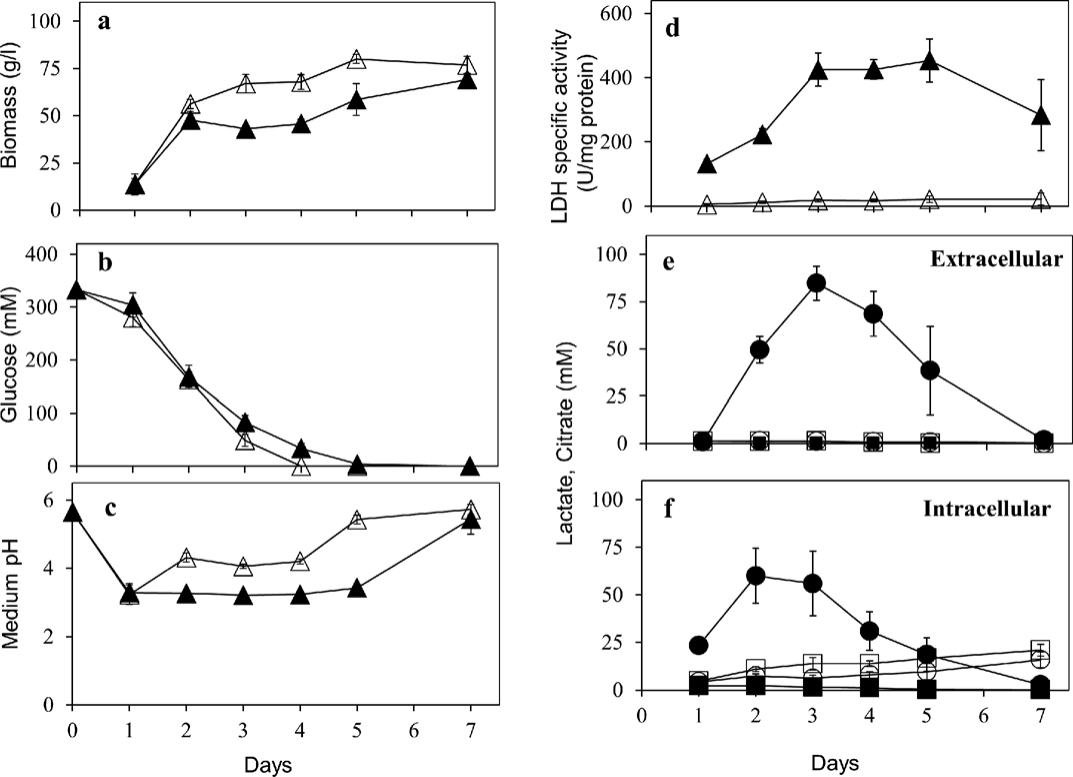
Lactate formation from glucose by *A*. *niger* C12 strain: The shake flask growth over 7 days was performed and biomass (Panel a; wet weight), medium glucose (Panel b), medium pH (Panel c), LDH specific activity (Panel d) and extracellular (Panel e) and intracellular (Panel f) concentrations of the two acids are shown. In panels a, b, c and d, filled triangles represent data for C12 strain while open triangles represent data for the parent strain. The symbols in panels e and f mean the same as in Fig 4.

An extended time course of lactate formation by C12 strain was monitored by growth on MM supplemented with 60 g/l of glucose. While their glucose consumption was comparable (Fig 5B), the C12 strain formed less biomass than the parent strain during the lactate production phase (day 2 to day 4; Fig 5A). The LDH activity in C12 strain was detected throughout the seven days of growth (Fig 5D). The L-lactate levels and LDH specific activity (as also the total LDH activity) peaked on day three. By this time, C12 strain produced 85 mM (7.7 g/l) of extracellular L-lactate from 333 mM (60.0 g/l) of glucose; with an overall yield of 13 percent (g lactate/g glucose) (Fig 5E). The pH of the spent medium decreased to around 3.0 (Fig 5C) upon lactate accumulation. This may not be an issue as *A*. *niger* is tolerant to and capable of growth in acidic pH particularly during acidogenesis [23]. However, lactate production by engineered yeasts is adversely affected in non-neutralizing media; addition of CaCO_3_ significantly improved the lactate yield [10,12,36]. Therefore, lactate secretion by C12 strain was also monitored under neutralizing conditions—on MM buffered with CaCO_3_ (30 g/l). The medium pH was stable between pH 6.0–6.3 during the course of fermentation; with a maximum extracellular lactate concentration of 70 mM on day four (not shown). While addition of CaCO_3_ in MM had no effect on lactate secretion by C12 strain, extracellular lactate did not disappear (see below) at later growth stages. Addition of CaCO_3_ had no significant effect on lactate titers and conversion yields in the case of *A*. *brasiliensis ldh* transformant. However, the nature of nitrogen source in the medium affected the lactate titers [22]. The recent report on lactate fermentation by engineered *A*. *oryzae* provides data only with CaCO_3_-supplemented media [21]. While CaCO_3_ could be added to production media, undesirable costs to downstream processing of lactate may accrue [1].

Intracellular lactate concentrations have been reported only in *C*. *sonorensis* engineered yeast [12]. Lactate concentrations inside C12 strain were comparable to that secreted in the non-neutralizing MM (Fig 5F). Nevertheless, intracellular lactate levels were invariably high at all the glucose concentrations tested (Fig 4). Lactate is known to be actively transported and hence could be a bottleneck. Over-expression of lactate permease (encoded by *JEN1*) improved lactate production by an engineered *S*. *cerevisiae* [37]. Presence of a functional lactate transporter (*lacA*) was also demonstrated in *R*. *oryzae* [44]. *A*. *niger* genome harbors a putative lactate transporter gene (NCBI accession number CAK42727), which is homologous to *JEN1* of yeast. Engineering a functional lactate transporter might augment lactate secretion in *A*. *niger*.

Since *A*. *niger* is acid tolerant and a prolific citrate producer [23], a comparison of lactate and citrate formation by the C12 strain was of interest. Both *A*. *brasiliensis and A*. *niger* belong to black Aspergilli but the effects on citrate levels due to *ldhA* expression in the former are not known [22]. L-Lactate levels were always far higher than citrate levels of C12 strain (Fig 5E and 5F). Intracellular citrate in C12 strain remained largely steady whereas extracellular citrate levels were very low. This pattern of citrate levels (both extracellular and intracellular) was comparable to that of the parent strain under all growth conditions tested (Figs 4, 5E and 5F). Therefore, inducing lactate formation by *mldhA* expression does not appear to perturb citrate levels in *A*. *niger*. This is an advantage in lactate fermentation in MM with minimal interference of citrate.

#### Lactate fermentation on AM

Citric acid fermentation conditions are well established in *A*. *niger*. Inducing lactate formation by *mldhA* expression in C12 strain does not appear to influence citrate levels when grown in MM. This was tested further by growing C12 strain in AM (conducive to citrate production). Despite the presence of active LDH throughout the course of fermentation, both extracellular and intracellular lactate levels in the C12 strain were negligible (Table 4). However, both parent and C12 strain of *A*. *niger* displayed high citrate levels. Citrate synthase activity is present throughout the acidogenic growth of *A*. *niger* [23]. In the present study, citrate synthase promoter was employed to express mouse *ldhA* [24]. It is interesting that minimal lactate was produced by the C12 strain grown on AM. Pyruvate availability may be a factor as pyruvate carboxylase is also active during acidogenesis [23].

**Table 4:** LDH specific activity, lactate and citrate levels in *A*. *niger* grown on AM.

Strain	LDH specific activity (U/mg protein)	Lactate (mM)		Citrate (mM)	
		Extracellular	Intracellular	Extracellular	Intracellular
C12	128.5 ± 29.0	0.23 ± 0.10	0.85 ± 0.25	9.7 ± 4.8	2.7 ± 1.1
Parent	4.25 ± 1.8	0.02 ± 0.01	0.20 ± 0.14	16.0 ± 1.0	5.0 ± 1.3

LDH activity and lactate/citrate was estimated after eight days of growth.

#### Lactate fermentation under hypoxic and nitrogen starvation

The conversion of glucose to L-lactate by pre-grown mycelia of C12 strain was assessed under hypoxic conditions and on MM devoid of nitrogen source. After 48 h incubation in these two conditions, no significant increase in biomass was observed whereas extracellular lactate levels remained around 5 mM. In a similar study, lactate yield by the parent strain was negligible (10–15 μM). Lactate formation in C12 strain therefore appears to be a growth associated phenomenon.

Among all growth conditions tested, maximum lactate secretion by C12 strain was obtained on non-neutralizing MM with 6 percent glucose. Lactate was secreted within 3 days of fermentation which is less compared to 4 and 10 days required by engineered *A*. *brasiliensis* and *A*. *oryzae*, respectively (Table 5). Although the lactate yield obtained was low, it is comparable to other engineered fungi grown on non-neutralizing media (Table 5). Further improvements can be achieved through media and growth optimization. More interestingly, lactate and citrate production could be separated based on growth on two different media. Exploring lactate production by C12 strain on other carbon sources also merits further attention.

**Table 5:** L-Lactate production by engineered fungi.

Fungus^a^	Source of *ldhA*	Glucose (g/l)	Lactate (g/l)	Fermentation time (days)	Reference
*S*. *cerevisiae* (Δ*pdc*1Δ*adh*1)	Bovine	100	74.0	2	[45]
*K*. *lactis* (Δ*KlPDC*1)	Bovine	50	12.0	4	[7]
*C*. *utilis* (Δ*CuPDC1*)	Bovine	110	103.3	1.5	[11]
*C*. *sonorensis* * (Δ*pdc1*Δ*pdc2*)	*L*. *helveticus*	50	10.0	5	[12]
	*B*. *megaterium*	50	5.0	5	[12]
	*R*. *oryzae*	50	4.0	5	[12]
*P*. *stipites* *	*L*. *helveticus*	55	8.0	2	[10]
*A*. *oryzae (*Δ871*)*	Bovine	100	45.0	10	[21]
*A*. *brasiliensis* *	*R*. *oryzae*	50	13.9	5.5	[22]
*A*. *niger* *	Mouse	60	7.7	3	This study

^a^ Respective gene deletions are shown in the brackets.

* Growth was carried out in non-neutralized medium.

### Lactate utilization by *A*. *niger* C12 strain

Very little is known about lactate metabolism in Aspergilli [46]. *A*. *niger* was unable to utilize and grow on L-lactate as sole carbon source, both on liquid and solid MM. Expression of a functional LDH was expected to support the growth of C12 strain on L-Lactate; however, this was not the case. Besides unfavorable kinetic features of mouse LDH for lactate oxidation, uptake of lactate could be an issue. Efforts to feed lactate at lower pH values (pH 3.0 and pH 5.7) and also as methyl lactate were unsuccessful. The C12 strain (as well as the parent strain) grew very poorly on other three-carbon compounds like L-alanine, glycerol or glycerol plus lactate. *A*. *niger* is incapable of utilizing ethanol as a carbon source [32]. An inefficient gluconeogenesis in *A*. *niger* is one possibility. Interestingly, pre-formed intra and extracellular lactate levels decreased on prolonged growth of C12 strain in non-neutralizing MM (see Fig 5E and 5F). Disappearance of lactate only after glucose exhaustion suggests its reutilization by this strain. Such a lactate utilization/ reutilization possibility was not examined in studies with *A*. *oryzae* and *A*. *brasiliensis* [21,22]. Both C12 and parent strains were fed with additional lactate (20 mM) after 5 days of growth on MM (6 percent glucose, without pH buffering). Surprisingly, decrease in added lactate was observed for both the parent and the C12 strain within 24 h of further growth. These results point to utilization of lactate through an endogenous enzyme of the parent strain (and not via the expressed mouse LDH). However a mechanism for the observed lactate utilization in *A*. *niger* (Fig 5E and 5F) remains to be elucidated. Extracellular lactate decreased after glucose consumption, both in *S*. *cerevisiae* [47] and *C*. *sonorensis* [12]. It was proposed that such lactate reutilization may be a strategic response to weak acid stress. *S*. *cerevisiae* utilizes L-lactate via a mitochondrial L(+)-lactate:cytochrome c oxidoreductase (encoded by *CYB2*) and disruption of *CYB2* improved the lactate production [48]. Lactate yield marginally improved in *A*. *oryzae* when a putative *ldh* (AO090023000871) locus was disrupted [21]. Putative NAD- and cytochrome-dependent *ldh*(s) are also known in *A*. *niger* and these need further attention.

## Conclusions

The high acidogenic potential and glycolytic flux make *A*. *niger* an attractive target for the production of lactic acid. Its genome was analyzed for potential *ldhA* genes for functional expression. While *A*. *niger* may contain an endogenous LDH (or LDH-like) activity, an *ldhA* gene coding for such an activity could not be identified. Considering the metabolic features of the *A*. *niger* pyruvate node, the LDH expression construct was designed based on sequence comparisons and using an endogenous promoter relevant to its acidogenic metabolism. The integration of *PcitA*-*mldhA* DNA led to LDH expression and resulted in transformants that gave significant lactic acid titers. Lactate formation was analyzed as a function of different growth media, hypoxia and nitrogen starvation. Although *A*. *niger* does not utilize lactate as a sole carbon source, it was able to re-utilize secreted lactate subsequent to glucose consumption. Interestingly, the formation of lactate and citrate by the C12 strain could be segregated by choosing different growth media. Already an industrial favorite, *A*. *niger* promises to be a good platform to produce lactate as yet another organic acid.

## Supporting Information

S1 Fig Multiple sequence alignment of LDH sequences from different organisms. The relevant sequence alignment region covering the LDH active site signature (underlined) is shown.
(TIF)

S1 Table QPCR data for determining *PcitA-mldhA* copy number in *A*. *niger mldhA* transformants. (DOC)
