# Supplementary figures and images for: Expression of Lactate Dehydrogenase in Aspergillus niger for L-Lactic Acid Production
Source: PLoS One. 2015 Dec 18;10(12):e0145459. doi: 10.1371/journal.pone.0145459 (PMC4684279; doi:10.1371/journal.pone.0145459)

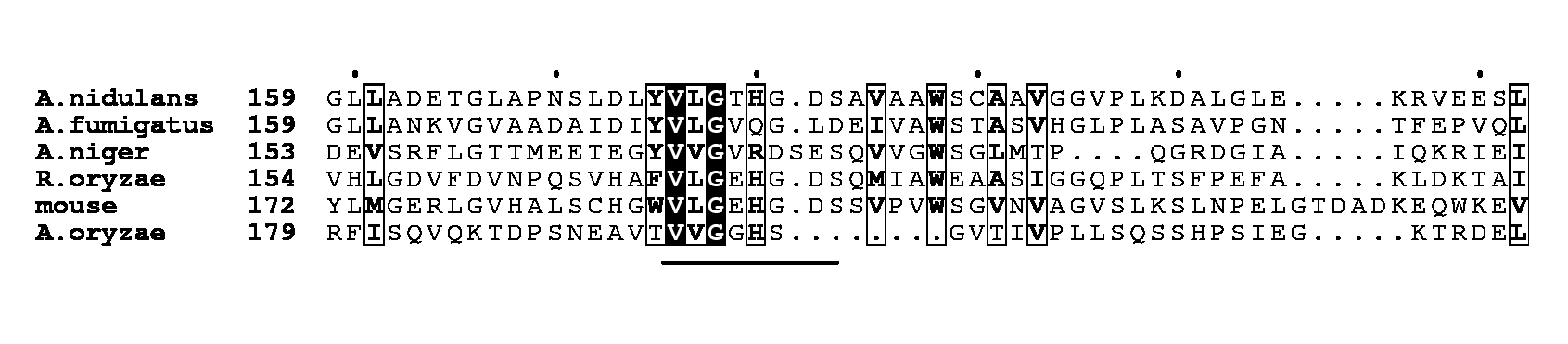

Supplement: S1 Fig — The relevant sequence alignment region covering the LDH active site signature (underlined) is shown. (TIF) [file pone.0145459.s001.tif]
