# Supplementary material for: Expression of Lactate Dehydrogenase in Aspergillus niger for L-Lactic Acid Production
Source: PLoS One. 2015 Dec 18;10(12):e0145459. doi: 10.1371/journal.pone.0145459 (PMC4684279; doi:10.1371/journal.pone.0145459)
Supplement: S1 Table — (DOC) [file pone.0145459.s002.doc]

**Supplementary data**

**Table S1**: QPCR data for determining *PcitA-mldhA* copy number in *A. niger mldhA* transformants

| ***A. niger* strains** | **Ct^a^ value at different DNA concentration (ng/µl)** | | | |
| --- | --- | --- | --- | --- |
|  | **0.1** | **0.5** | **1** | **10** |
| D5 | 20.29 | 17.85 | 16.76 | 13.89 |
| C12 | 19.65 | 16.84 | 15.87 | 12.58 |
| C2 | 18.42 | 16.25 | 15.10 | 12.06 |
| C7 | 22.00 | 19.56 | 18.81 | 15.90 |
| C16 | 23.49 | 20.87 | 20.05 | 16.95 |
| C3^b^ | - | - | - | 29.13 |
|  |  |  |  |  |
| Parent^c^ | 23.84 | 21.19 | 19.99 | 16.61 |

^a^ Ct values were average of triplicate reactions

^b^ Ct value was comparable to “no template control”

^c^ Ct values are for *A. niger actA* gene
